# Bioethanol Production from Lignocellulosic Biomass—Challenges and Solutions

**DOI:** 10.3390/molecules27248717

**Published:** 2022-12-09

**Authors:** Magdalena Broda, Daniel J. Yelle, Katarzyna Serwańska

**Affiliations:** 1 Department of Wood Science and Thermal Techniques, Faculty of Forestry and Wood Technology, Poznań University of Life Sciences, Wojska Polskiego 28, 60-637 Poznan, Poland; 2 Forest Biopolymer Science and Engineering, Forest Products Laboratory, USDA Forest Service, One Gifford Pinchot Drive, Madison, WI 53726, USA; 3 Department of Animal Anatomy, Faculty of Veterinary Medicine and Animal Sciences, Poznan University of Life Sciences, Wojska Polskiego 71c, 60-625 Poznan, Poland; 4 Department of Sports Dietetics, Poznan University of Physical Education, 61-871 Poznan, Poland

**Keywords:** bioethanol, ethanol, lignocellulose, lignocellulosic materials, lignocellulosic biomass, lignocellulosic complex, fermentation, biomass utilisation, biofuel, green fuel, biorefinery

## Abstract

Regarding the limited resources for fossil fuels and increasing global energy demands, greenhouse gas emissions, and climate change, there is a need to find alternative energy sources that are sustainable, environmentally friendly, renewable, and economically viable. In the last several decades, interest in second-generation bioethanol production from non-food lignocellulosic biomass in the form of organic residues rapidly increased because of its abundance, renewability, and low cost. Bioethanol production fits into the strategy of a circular economy and zero waste plans, and using ethanol as an alternative fuel gives the world economy a chance to become independent of the petrochemical industry, providing energy security and environmental safety. However, the conversion of biomass into ethanol is a challenging and multi-stage process because of the variation in the biochemical composition of biomass and the recalcitrance of lignin, the aromatic component of lignocellulose. Therefore, the commercial production of cellulosic ethanol has not yet become well-received commercially, being hampered by high research and production costs, and substantial effort is needed to make it more widespread and profitable. This review summarises the state of the art in bioethanol production from lignocellulosic biomass, highlights the most challenging steps of the process, including pretreatment stages required to fragment biomass components and further enzymatic hydrolysis and fermentation, presents the most recent technological advances to overcome the challenges and high costs, and discusses future perspectives of second-generation biorefineries.

## 1. Introduction

Concerning the continuously increasing global demand for energy, fossil fuel resources on our planet are anticipated to become depleted within the next several decades, endangering worldwide energy security. More importantly, the combustion of fossil fuels contributes to CO_2_ emissions and hence global warming, a rise in sea levels, urban pollution, and loss of biodiversity, constituting a threat to the global environment. Therefore, the energy transition to low-carbon-intensity fuels becomes necessary to tackle climate change [1,2].

**Figure 1:**
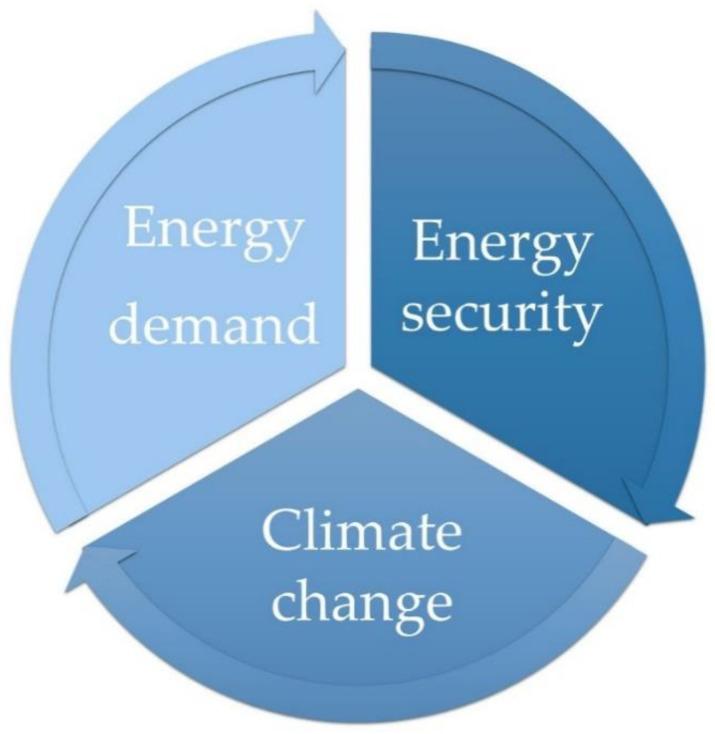
Three main reasons to develop the production of biofuels.

All these negative environmental, social, political, and energy security concerns of the current world has boosted interest in alternative energy sources, including biofuels. However, although alternative energy sources hold the key to solving the three critical global problems, i.e., energy demand and security and climate change (Figure 1), the transition from fossil fuels to more sustainable energy resources require a high initial investment and innovative technologies. Therefore, employing an energy mix of fossil fuels, biofuels, and renewable energy sources seems to be a good starting strategy to switch to solely sustainable resources in the near future [1,2,3,4].

Biofuels emerged as a promising alternative to fossil fuels [5,6,7]. Among them, bioethanol is one of the most attractive as it can substitute gasoline [8,9,10,11]. As a result, several countries, including the USA, Brazil, China, Canada, India, Thailand, Argentina, and many EU members, have already proclaimed commitments to reducing their dependence on fossil fuels towards developing bioethanol production. However, industrial-scale bioethanol production still faces a severe challenge of suitable feedstock acquisition and economic viability while environmentally friendly production technology [1,12,13].

Bioethanol can be produced from a variety of renewable materials rich in carbohydrates, which can be hydrolysed to fermentable sugars and converted to ethanol. Three main feedstock types can be used for bioethanol production: sucrose and starch crops, such as cereals, sugarcane, corn, and others similar (the first-generation bioethanol) [14,15,16], lignocellulosic biomass (the second-generation bioethanol) [12,17,18], and microalgae (the third-generation bioethanol) [19,20,21].

**Figure 2:**
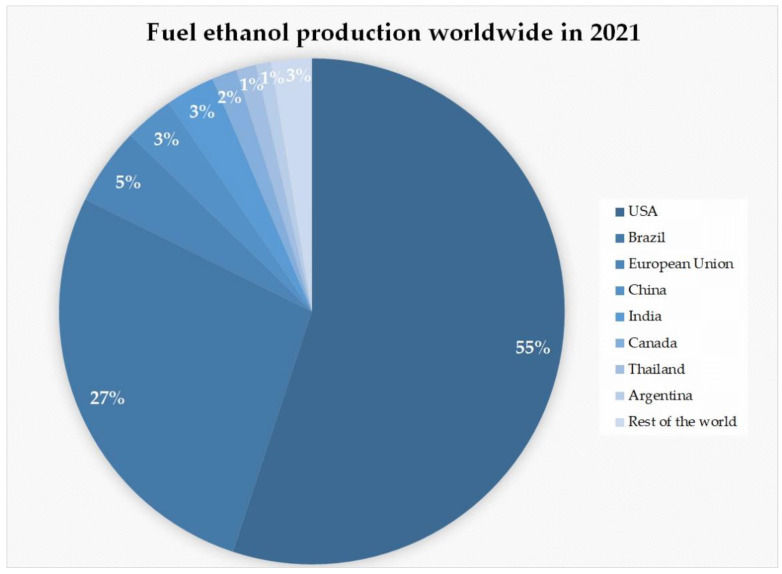
World’s top leaders in bioethanol production in 2021 (based on [22]).

The first-generation bioethanol constitutes the majority of over 27,000 million gallons (over 102,060 million litres) of bioethanol produced worldwide (status as of 2021), with the United States of America and Brazil being the indisputable leaders producing almost 85% of the global output mainly from corn and sugarcane, respectively (Figure 2) [22]. On the other hand, France and Germany are the leading bioethanol producers in Europe (Table 1). The primary feedstock for bioethanol production is wheat (in Belgium, Germany, France, and the UK), corn (in Central Europe, the Netherlands, and Spain), sugar beets (in France, Germany, the UK, the Czech Republic, Belgium, and Austria), as well as beet pulp or concentrated juice (in Austria and Belgium) [23]. However, the increasing bioethanol production levels, along with the growing population, raise concerns over the long-term sustainability of first-generation bioethanol, including a threat to global food and feed security, demand for land and water resources, and potential contamination of soil with distillation residues, which prompts intensive research on alternatives, such as second- and third-generation bioethanol production technologies [14,24,25].

Over the past few decades, the experience gained from first-generation bioethanol production has paved the way for new technologies enabling the utilisation of more sustainable feedstock without adverse effects on food supplies and the environment. Second-generation biorefineries are based on widely available lignocellulosic biomass generated from various sectors (see Section 2.1) that are not directly used as food, and new technologies to convert the biomass into ethanol and other valuable co-products have been continuously developed. Such an approach has the potential to meet energy demands sustainably in an economically viable and environmentally safe way [13,14].

The existing or planned second-generation biorefineries in the US with their production capacity are listed in Table 2. Total cellulosic ethanol production for 2022 in Brazil is estimated at 55 million litres, with an increase of 15 million litres compared to 2021 [26]. In the European Union, there are only a few advanced biofuel plants producing second-generation bioethanol at a commercial scale (Table 3); several others, based on sawdust, forest residues, cereal straw, and by-products from cellulose production, are planned to be opened soon in Finland, Norway, Slovakia, Romania, and Austria [23]. However, commercial production of second-generation bioethanol still represents only an insignificant share of total ethanol production worldwide.

Second-generation bioethanol has gained increased interest from governments, large companies, and academic research over the past two decades since it represents an attractive renewable alternative to diminishing fossil fuels. Its production is also widely accepted by the general public as it is perceived as non-competitive with the food and feed market and can help mitigate climate change. However, the commercial production of cellulosic ethanol is still in its infancy, being hampered by the high cost of research and production, and tremendous efforts are required to make it more widespread and profitable [17,28,29].

In this paper, we present the state of the art in bioethanol production from lignocellulosic biomass, discuss the most challenging stages of the process, highlight the up-to-date solutions and technological advances that can increase the efficiency of fuel ethanol yield and reduce production costs, and debate future perspectives of second-generation biorefineries.

## 2. Production of Bioethanol from Lignocellulosic Biomass

**Figure 3:**
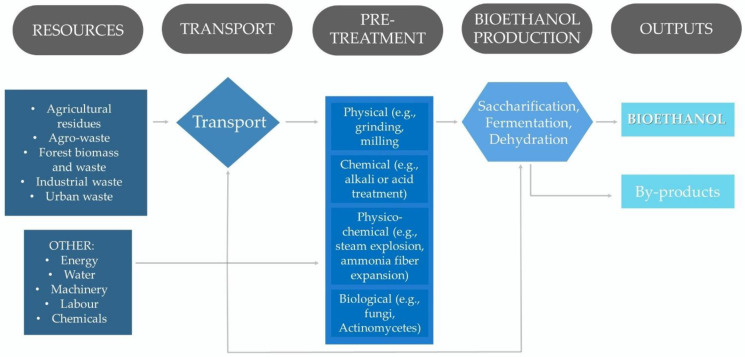
Five elements of bioethanol production, including resources, transportation, all stages of processing and conversion technologies with associated inputs, and final products (based on [3,30]).

Bioethanol production from lignocellulosic biomass is a complex and lengthy process. It includes several steps from resources to end products, such as sourcing of raw materials (lignocellulosic biomass) and their transportation, biomass pretreatment, saccharification, fermentation and ethanol dehydration, products and by-products management, plus all other resources necessary for the production process, including labour, machinery, utilities, and chemicals (Figure 3).

Although biomass transportation and the utilisation of other resources such as energy, chemicals, labour, machinery, and water are essential and often challenging elements in ethanol production, we omit them in this paper to focus primarily on the processes directly related to the conversion of lignocellulosic biomass into bioethanol, namely pretreatment, hydrolysis, and bioethanol release, and on the associated challenges and new solutions.

### 2.1. Lignocellulose Resources

Lignocellulosic biomass (or lignocellulose) refers to all plant dry matter (biomass) on Earth, which is the most abundant renewable raw material. Due to the presence of fermentable components (carbohydrates, cellulose, and hemicelluloses), lignocellulose is one of the alternative feedstocks for bioethanol production [1].

**Figure 4:**
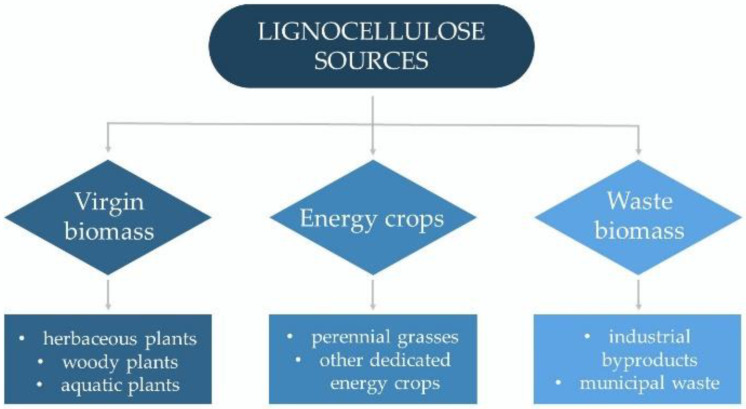
Sources of lignocellulosic biomass for bioethanol production.

Lignocellulose resources can be broadly classified into three main groups: virgin biomass, energy crops, and waste biomass (Figure 4). Virgin biomass comprises all naturally growing terrestrial plants, including herbaceous plants (annual, biennial, and perennial plants) and woody plants (trees, bushes, and dwarf shrubs), as well as aquatic plants (e.g., water hyacinth, water fern, water lettuce, and duckweed). Energy crops include perennial grasses and other dedicated energy crops that produce a high yield of lignocellulosic biomass (e.g., switchgrass, giant reed, elephant grass, and miscanthus). Waste biomass is a low-value by-product of different industrial sectors such as agriculture (bagasse, cereal straws, stover, and husks), forestry (branches from dead trees, pruning, and thinning residues), and wood and paper production (bark, sawdust, and wood chips). It also includes an organic portion of municipal solid wastes [1,31,32].

Each group of lignocellulose sources has some potential to serve as a raw material for bioethanol production. However, their usability depends on the polysaccharide content that varies between the type of biomass, plant species, and individual parts of the plant. Generally, lignocellulosic biomass is the worldwide most abundant feedstock for ethanol production and has numerous advantages: it is cost-efficient, it is readily available, it does not interfere with food and feed production, it does not require any extra land, and it provides a continuous and reliable supply. Additionally, its utilisation for bioethanol production lessens the problem of waste biomass management and fits in with a sustainable, environmentally-friendly, zero-waste circular economy [1,13,33].

#### The Structure of the Lignocellulosic Complex

**Figure 5:**
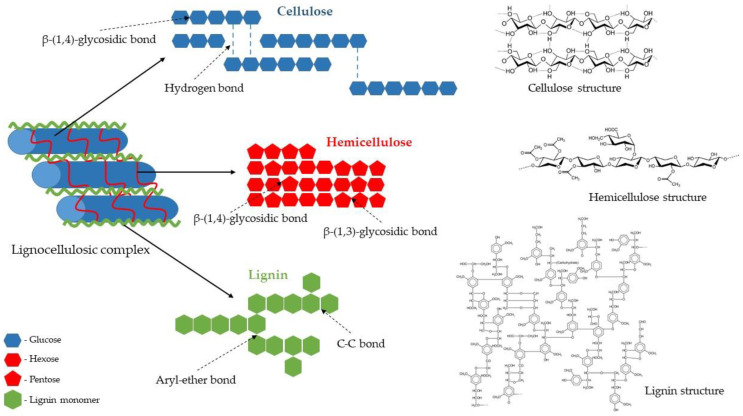
A schematic structure of the lignocellulosic complex (based on [35,36]).

Lignocellulose is the main structural component of plant cell walls. It is composed of three different polymers: polysaccharides (cellulose and hemicelluloses) and lignin, a complex aromatic polymer synthesised via radicals of hydroxycinnamyl alcohols (Figure 5). The amount of individual polymers vary depending on the biomass origin (e.g., plant species and part of the plant). However, cellulose and hemicelluloses usually constitute about two-thirds of its total dry mass [1,34].

Cellulose is the main structural polysaccharide of the plant cell wall, providing it with high tensile strength and rigidity. Its amount usually ranges from 30% to 50% of the dry weight of lignocellulosic biomass. Regardless of its origin, cellulose is generally a highly crystalline and a high-molecular-weight polymer with a strong tendency to form high-crystalline fibres. A cellulose molecule is a long-chain straight linear homopolysaccharide with an average molecular weight of about 100,000 Da. It consists of β-D-glucopyranose units linked by β-1,4-glycosidic bonds, with the disaccharide cellobiose, made of two β-glucose molecules connected by a β(1→4) bond, as its repeating unit (Figure 5). Owing to the presence of reactive hydroxyl groups at C6, C2, and C3 of glucopyranose units, an extensive network of intra- and inter-chain hydrogen bonds is formed. It induces crystalline structure and facilitates the organisation of the individual cellulose chains into bundles (microfibrils) that are additionally stabilised by van der Waals interactions (hydrophobic interactions), leading to a fibrous state. Highly ordered crystalline regions in cellulose are interspersed with disordered amorphous regions. The explicit cellulose structure makes it insoluble in water and resistant to depolymerisation [1,37,38].

Hemicelluloses are, collectively, the second most abundant polysaccharide of the plant cell wall, which accounts for 15–30% of the lignocellulosic dry mass. They are complex polymers consisting of short linear and highly branched heteropolysaccharides (Figure 5) with an average molecular weight of about 30,000 Da, consisting of various sugar units, both pentoses (β–D–xylose and α–L–arabinose) and hexoses (β–D–glucose, α–D–galactose, and β–D–mannose), as well as uronic acids (α–D–glucuronic, α–D–galacturonic, and α–D–4–*O*–methylgalacturonic acid), and minor amounts of α–L–rhamnose and α–L–fructose. Among heteropolymers present in hemicelluloses are the most common xylan and glucomannan, as well as glucuronoxylan, arabinoxylan, and xyloglucan. This composition makes hemicelluloses’ structure random and amorphous, or only partially crystalline. Hemicelluloses are embedded in the plant cell walls, and their segments bind with adjacent cellulose microfibrils through hydrogen bonding and van der Waals interactions. This cellulose–hemicellulose network acts as a load-bearing element that strengthens the cell wall [1,39,40,41].

Lignin is the third principal component of lignocellulosic biomass, constituting about 15–30% of its dry mass. Present in all vascular plants, lignin is an amorphous, hyperbranched, and a cross-linked three-dimensional network polymer with no regular repeating elements (Figure 5). This random structure arises due to the enzymatically initiated free radical polymerisation of three types of phenylpropane units (also known as monolignols) such as *p*-coumaryl, coniferyl, and sinapyl alcohols that form aromatic units of lignin: *p*-hydroxyphenyl, guaiacyl, and syringyl, respectively. The proportion of monomers forming the lignin molecule differs depending on the type of plant, cell, and location in the cell wall structure. For example, hardwood lignin is made up mainly of coniferyl and sinapyl alcohols (guaiacyl-syringyl lignin and GS-lignin), softwood lignin is made of coniferyl alcohol (guaiacyl lignin and G-lignin), while lignin of grass and herbaceous plants contains coniferyl, sinapyl, and *p*-coumaryl alcohol (*p*-hydroxyphenyl-guaiacyl-syringyl lignin and HGS-lignin). Lignin is covalently bonded to hemicelluloses and part of cellulose, serving as a cement between the fibres, supporting the mechanical properties of the cell walls, and protecting the structural polysaccharides from enzymatic microbial degradation [1,42,43,44].

Each of the main cell wall structural polymers has a different chemical structure and, thus, behaviour. Moreover, they are strongly intertwined and bonded by non-covalent forces (hydrogen bonds and van der Waals interactions) and covalent cross-linkages, forming a highly structured and robust composite matrix. All these, together with a crystalline cellulose nature and low surface area available for enzymes, make lignin complex and highly recalcitrant and resistant to separation and depolymerisation. Therefore, processing lignocellulosic biomass for bioethanol production is challenging, requiring proper knowledge and technologies that employ a combination of chemicals, enzymes, microorganisms, and heat [1,12,34,45].

Among lignocellulose structural polymers, only cellulose and hemicelluloses can be used to produce bioethanol because they are long-chain polysaccharides hydrolysed into a mixture of fermentable pentoses and hexoses that can be further converted to ethanol molecules. However, owing to the recalcitrance of lignocellulosic biomass, obtaining high efficiency and profitability in the process of bioconversion of lignocellulosic substrate into ethanol requires a primary pretreatment process. It is necessary to first release cellulose and hemicelluloses from a complex matrix and make them more accessible towards enzymatic hydrolysis [28,45,46].

### 2.2. Pretreatment of a Lignocellulosic Biomass

The pretreatment of the lignocellulosic complex is the first and necessary step in its bioconversion to ethanol. During this process, the structure of lignocellulose is disrupted by breaking down cross-linkages between its structural polymers, which helps to separate carbohydrates from lignin, and hydrogen bonds between cellulose chains are broken, thus decreasing cellulose crystallinity and its degree of polymerisation. Pretreatment technologies are intended to improve the accessibility of enzymes to carbohydrates by reducing the size of biomass particles and boosting their surface area and porosity, thus facilitating their hydrolysis and fermentation; they also increase yields of fermentable sugars [45,47,48].

The efficiency of the polysaccharide hydrolysis to monosaccharides, the primary substrates in alcoholic fermentation, is mainly limited by the presence of lignin. On the one hand, the lignin polymer restricts the free access of hydrolytic enzymes to cellulose microfibrils and hemicellulose chains. On the other hand, lignin acts as an adsorbent that binds the enzyme molecules on its surface, thus causing their irreversible inactivation. Therefore, lignin has to be removed in the pretreatment step [1,3,45]. Other limiting factors are inhibitory compounds that can be produced during the pretreatment stage, including furan derivatives (HMF–5-hydroxy-2-methyl-furfural and furfural), phenolic compounds, and weak acids (acetic, formic, and levulinic acid). They adversely affect hydrolysis efficiency by limiting microbial activity and/or disturbing enzymes’ efficiency; therefore, their presence is highly undesirable [45].

The pretreatment process should be easy to carry out, cost-effective, and environmentally friendly, producing minimum amounts of inhibitory compounds and allowing a complete utilisation of lignocellulosic biomass, which results in high efficiency of bioethanol production and the proper management of waste lignin. Generally, the existing pretreatment methods can be grouped into four categories, such as physical, chemical, physico-chemical, and biological (Figure 1). Unfortunately, there is no universal pretreatment for all types of biomasses and, usually, a combination of two or more complementary techniques is applied to obtain the most satisfactory results. However, developing the best pretreatment strategies is still a subject of extensive research [45,47,48,49].

#### 2.2.1. Physical Pretreatment

Physical pretreatment methods employ mechanical forces, irradiation, electric or electromagnetic field, temperature, or pressure to reduce the size of lignocellulosic biomass particles and increase their surface area and pore volume. They usually also decrease the degree of all components’ polymerisation and cellulose crystallinity, which facilitates further biomass processing. The physical methods include grinding, milling, chipping, extrusion, freezing, sonication, microwaving, and pulsed electric field treatment [45,47].

Chipping, grinding, and milling are the primary pretreatment techniques to crush lignocellulosic biomass. Depending on the method, the final particle size can be reduced to 10–30 mm or even 0.2–2 mm. Among the mechanical methods, ball milling, colloid milling, hammer milling, two-roll milling, and wet disk milling are commonly used in bioethanol production, with ball milling giving the highest yields of glucose and xylose after enzymatic hydrolysis [45,47,50]. However, milling is relatively expensive due to high energy requirements [45,47].

Extrusion is a thermo-physical method that includes rapid mixing, moderate heating, and high shearing of lignocellulosic biomass, resulting in physical and chemical disruption of its complex structure. The process is highly versatile and efficient, does not produce furfural and HMF, and can be carried out continuously, even for high solids loading. However, due to high energy requirements, it may not be economically the best alternative to conventional pretreatment [45,51,52].

Freeze pretreatment is a relatively new and promising technique. It was shown to significantly increase the enzymatic conversion of rice straw, resulting in enhanced glucose and bioethanol yields. In addition, this method has a low environmental impact and is relatively cost-effective due to the low energy input required and the lack of toxic chemicals involved in the process [53,54].

Sonication employs ultrasound waves to disrupt the lignocellulose complex and make cellulose and hemicelluloses available for enzymatic hydrolysis. As a result, the method enhances the conversion of cellulose to fermentable sugars, increases sugar yields, reduces hydrolysis time, and improves further fermentation. The application of slightly elevated temperatures (about 50 °C) and a change of water into an alkaline medium can additionally ameliorate the pretreatment process [47,55].

Microwave irradiation penetrating through the lignocellulosic feedstock effectively disrupts its recalcitrant structure. This can improve the solubilisation of lignocellulosic biomass, effectively degrade lignin, and alter the structure of the polysaccharides, thus enhancing their susceptibility to hydrolysis. Using higher power and temperatures increases the effectiveness of the process. Microwave-assisted pretreatment can be an interesting alternative to conventional heating due to its uniformity and selectivity, less energy input, and shorter processing time [45,47,56].

Pulsed electric field (PEF) treatment is a novel method that increases biomass porosity and permeability by subjecting it to a series of high voltage (5.0 and 20.0 kV/cm) short-duration (nano to milliseconds) pulses. The technique seems to be cost-efficient due to low energy requirements and the simplicity of instrumentation required that can be easily designed to the biorefinery conditions, and it enhances cellulose hydrolysis resulting in its efficient conversion to bioethanol [47,57].

#### 2.2.2. Chemical Pretreatment

Chemical pretreatment employs various chemicals, including alkalis, acids, gases, salts, ionic liquids, oxidising agents, or organic solvents, to release polysaccharides from the lignocellulosic complex and make them more susceptible to enzymatic hydrolysis [3,45,47].

Acid pretreatment is one of the most frequently used methods to overcome the recalcitrance of lignocellulose in bioethanol production. Biomass is usually treated with mineral acids solutions (HCl and H_2_SO_4_) at a pressure of 1.5 bar and elevated temperatures ranging from 100 °C to 290 °C for various residence times (up to several hours). The crucial effective parameters for the method include acid concentration, solids loading, temperature, and residence time. When diluted acids are used in the process, their effectiveness is enhanced by increasing the temperature of the process. Acid pretreatment has only a limited effect on lignin while mainly affecting polysaccharides. Hemicelluloses are dissolved and polysaccharide–lignin linkages are broken, thus making cellulose more accessible to enzymes. Its main disadvantages are the high cost of acid recovery and the production of inhibitory by-products. However, the environmental and economic aspects of the method have been improved recently [3,45,47,56].

In alkaline treatment, dilute solutions of NaOH, KOH, Ca(OH)_2_, or ammonia are usually used to degrade and remove lignin and part of hemicelluloses to make cellulose more available for enzymatic hydrolysis. By breaking crosslinks between hemicellulose and other polymers, the treatment also causes swelling of fibrous cellulose increasing biomass porosity. The process can be performed at elevated temperatures for a short time or at low temperatures for a relatively long period. The advantages of the method are selective lignin removal without a loss of carbohydrates and enhanced porosity of feedstock that improves further enzymatic hydrolysis, as well as biomass disinfection. The main drawback is longer reaction times (several hours up to one day) than other pretreatment methods [3,45,47,56,58].

Solvent pretreatment methods include the application of organic solvents, ionic liquids, and deep eutectic solvents. Several chemicals have been tested in this technique, such as acetone, ethanol, ethylene glycol, glycerol, methanol, n-butanol, phenol, tetrahydrofurfuryl alcohol, and triethylene glycol [3,45,47].

Organic solvent treatment employs a variety of organic solvents, including acetone, amines, alcohols, dioxane, esters, formaldehyde, propionic acid, and phenols with and without a catalyst, for the lignocellulosic biomass pretreatment. The technique is recognised as one of the most prospective pretreatment methods because of its ability to deconstruct lignocellulosic complex and fractionate biomass into lignin, cellulose, and hemicelluloses with high purity. It also allows for easy solvent recovery and reuse. Unfortunately, high energy consumption and the cost of organic solvents make the method not economically viable [47,59,60].

Ionic liquids (mainly salts including a large organic cation and small anion, including ammonium-based, imidazolium-based, phosphonium-based, pyridinium-based, pyrrolidinium-based, and sulfonium-based) have also been extensively studied for their potential to degrade lignin and break down crystalline cellulose structure. The method offers high rates of cellulose recovery and conversion to glucose. However, there are still many challenges to using ionic liquids on a broader scale, including their high price when large amounts are needed for the process, high waste generation with difficult recovery, high energy demands for recycling, and high viscosity of the solution over time that makes them difficult to handle [45,47,56,61,62].

A more “green” approach in bioethanol production involves biomass pretreatment with deep eutectic solvents. They are mixtures of hydrogen bond donors (e.g., amides, alcohols, or carboxylic acids) and acceptors (quaternary ammonium salts) at moderate temperatures of 60–80 °C, which enhance solubilisation of lignocellulosic polymers with higher selectivity towards lignin and without affecting cellulose.

Deep eutectic solvents are considered economical and “green” because they are less toxic than other chemicals used for conventional biomass pretreatment, easily biodegradable and recyclable, and have a great potential for much broader usage in the biorefineries of tomorrow [47,63,64].

The application of various metal salts for biomass pretreatment represents a more novel method that provides high sugar recovery, and its performance can also be further improved by combining it with other pretreatment technologies. The principal advantages of metal salt-based treatments are improved lignin removal, degradation of hemicelluloses, and complete biomass conversion. In addition, these pretreatments also result in enhanced enzymatic hydrolysis, are nontoxic and environmentally safe, and do not require costly non-corrosive reactors [47,65,66].

Another pretreatment method is biomass oxidation, which involves various oxidising agents, with hydrogen peroxide being the most frequently applied chemical. The method results in the degradation of lignin by hydroxyl radicals produced during hydrogen peroxide hydrolysis, which leaves the cell wall polysaccharides more accessible for further enzymatic hydrolysis. Since the method also degrades a part of hemicelluloses, it is not considered one of the most efficient processes for fermentation [47,67].

Ozonolysis is a greener oxidative pretreatment method that employs ozone gas as an oxidant to destruct the lignocellulose complex. Ozone reacts preferably with lignin, which results in effective biomass delignification and a release of sugar during enzymatic hydrolysis. The greatest advantage of this method is that it can be carried out in ambient conditions. Furthermore, the only inhibitory compounds produced are short-chain carboxylic acids, which can be easily removed by washing with water. However, the method is not economically viable due to the high costs of ozone since vast amounts are required in the process [45,68,69,70].

#### 2.2.3. Physico-Chemical Pretreatment

Physico-chemical methods utilise both physical (high temperature and pressure) and chemical processes to effectively pretreatment of lignocellulosic biomass. Among them, a steam explosion has been the primary technique used for bioethanol production. First, high temperature (160–260 °C) and pressure (0.7–4.8 MPa) are applied to biomass for a few seconds to several minutes; then, a sudden pressure reduction causes explosive decompression in the material. It results in the disruption of the cell wall structure and the solubilisation of hemicellulose and lignin fractions. A steam explosion is effective for all types of biomasses, including that with large particles, without a need for pre-crushing. The method’s main advantages are low energy requirements, no additional chemical costs (thus, no recycling), and environmental friendliness, while incomplete lignin removal and the production of some toxic chemicals during the process are the main disadvantages [3,45,47].

Ammonia fibre expansion (AFEX) applies liquid ammonia to lignocellulosic biomass under pressure and elevated temperature, followed by a rapid pressure reduction that expands the fibre structure, increasing its surface area. The treatment also causes the selective delignification of biomass, decrystallisation of cellulose, and partial hemicellulose depolymerisation, which results in high glucose yields in further enzymatic hydrolysis. Other AFEX advantages include that ammonia is a non-polluting and non-corrosive substance that can be easily recovered and reused in the process, and the amount that remains in the biomass serves as a nutrient source for microorganisms used in further bioethanol fermentation. The downside to this method is the small efficiency in the case of feedstock containing significant amounts of lignin [47,71,72,73].

Supercritical CO_2_ explosion is a green pretreatment method that employs supercritical fluid CO_2_ as a solvent. During its diffusion through the biomass under high pressure and temperature, carbonic acid is produced, which hydrolyses hemicelluloses. The subsequent explosion releases the gas that penetrated the structure of the lignocellulosic complex, thus weakening the cell wall ultrastructure and increasing the accessible surface area of its polymers for further enzymatic processes. CO_2_ necessary for the treatment can be sourced directly from glucose fermentation to ethanol, where it is released as a by-product and continuously recycled in the process without increasing CO_2_ emissions into the atmosphere. The method is environmentally friendly and efficient; it enhances glucose yield, facilitates biomass delignification, and allows the extraction of different components from the biomass; it is appropriate for all feedstocks that have retained some moisture. However, due to the costs of reactors suitable for high-pressure conditions, its application is limited [47,74,75,76].

Liquid hot water (LHW) pretreatment is a simple method that uses water under high pressure, similar to the steam pretreatment technique. Compressed water (at a pressure up to 5 MPa) at a high temperature (170–230 °C) permeates through lignocellulosic biomass, hydrolysing hemicelluloses, removing some lignin, and simultaneously hydrating the cellulose fraction, making it more accessible for enzymes. The method produces minimal inhibitory compounds. It is relatively cost-effective and environmentally friendly because it does not require an energy-demanding preliminary reduction in feedstock size and does not use chemicals or corrosion-resistant hydrolysis reactors [3,47,77].

For lignin-enriched feedstock, wet oxidation is a suitable pretreatment method that produces less inhibitory furan derivatives than a steam explosion or liquid hot water treatments. Oxygen or air is employed as a catalyst, and water or hydrogen peroxide serves as a medium. The process depends mainly on three critical factors, namely temperature, oxygen pressure, and time, and is typically carried out at a high temperature (above 120 °C) and pressure (0.5–2 MPa) for about 30 min. As a result, hemicelluloses undergo solubilisation and hydrolysis to monomers, and some lignin is oxidised, leaving cellulose more available for enzymatic processes. However, high financial expenditures imposed by oxygen and pressure equipment prices prevent the method from becoming the standard industrial application [45,47,78].

#### 2.2.4. Biological Pretreatment

Biological methods use ligninolytic microorganisms (bacterial and fungal strains) or their enzymes to reduce the recalcitrance of lignocellulosic biomass, converting it into compounds more accessible for hydrolysis and subsequent ethanol production. The most effective are white-rot fungi due to their ability to degrade lignin, including the four most frequently industrially used species: *Phanerochaete chrysosporium*, *Trametes versicolor*, *Ceriporiopsis subvermispora*, and *Pleurotus ostreatus*, and also some bacterial strains, including *Clostridium* sp., *Cellulomonas* sp., *Bacillus* sp., *Thermomonospora* sp., and *Streptomyces* sp., are commonly used in biological pretreatment. The crucial parameters affecting the efficiency of biological pretreatments are the type of selected microorganism, the size of biomass particles, and the process conditions, including moisture content, temperature, and time. The main advantages of biological pretreatment methods are their low energy requirements, no chemicals and their recycling costs, low downstream processing costs, the minimal amount of inhibitory compounds produced, relatively simple operating and environmental friendliness. However, the drawbacks, such as ample space requirement, a very slow course of the process, and the necessity of continuous control of microbial growth and activity, preclude more widespread application of these methods in the industry [45,47,56,79,80,81].

#### 2.2.5. Combined Pretreatment Methods

Apart from the pretreatment methods described above, there are various combinations and several other sophisticated techniques that are being developed to overcome the main drawbacks of the existing techniques to improve the utilisation of the lignocellulosic complex, making the bioethanol production process more economical, efficient and environmentally friendly [47,49,56].

For example, since the most common pretreatment methods require high temperatures (160–290 °C) and pressures (0.69 to 4.9 MPa), and additionally produce inhibitory furan derivatives, there is a need to develop new methods that overcome those disadvantages. One of them is alkaline hydrogen peroxide (AHP) pretreatment that combines the application of NaOH and H_2_O_2_. The main advantages are high effectiveness for various biomass concentrations providing high efficiency of enzymatic hydrolysis, high lignin and hemicellulose solubilisation values for the liquid fraction, low energy consumption, availability of chemicals needed, no furan derivatives produced, no need for special reactors, compatibility with high solid loadings, and sterility conditions provided by alkaline H_2_O_2_ without a need to use antibiotics. However, the method is not free from drawbacks, such as the high pH of pretreated biomass, the generation of other inhibitors, such as *p*-coumaric and ferulic acids, the price of chemicals required in high amounts, and the need for an initial grinding of the biomass material. To overcome these shortcomings, some specific approaches are still needed to optimise the whole pretreatment process and keep it efficient while being cost-effective and safe for the environment [82].

In biological pretreatment methods, a long operation time is one of the main disadvantages. To surmount this problem, extensive research has been conducted proposing to combine fungal treatment with various chemical, physical, or physico-chemical methods [83].

- Biological-alkaline pretreatment combination can enhance the delignification of a lignocellulosic complex and help reduce the chemicals’ concentration, time, and temperature of alkaline treatment, thus lowering operational expenses [83,84,85]. However, the treatment may cause a higher loss of carbohydrates from biomass [85].
- Biological-acid combination effectively solubilise the hemicellulose fraction and limit the production of inhibitory compounds while reducing severe acid pretreatment conditions. Moreover, it increases glucose and ethanol yield compared withacid pretreatment alone [83,84,86,87].
- Biological-oxidative pretreatment uses the fact that biomass decay by white-rot fungi involves a Fenton-based oxidation reaction. By mimicking this reaction using other oxidising reagents, e.g., hydrogen peroxide followed a biological pretreatment, it is possible to shorten the residence time and enhance biomass delignification without producing inhibitory by-products, which results in higher sugar yields. This combined pretreatment method seems to be the most effective among biological–chemical treatment combinations [79,83,88,89].
- Biological-organosolv combined pretreatment was studied for woody biomass, resulting in higher saccharification yields and larger amounts of lignin-enriched fractions [83,90,91].
- Biological-LHW treatment allows for the lowering of the temperature of the water during the LHW process while enhancing sugar yield owing to microbial activity [83,92,93].
- Biological-steam explosion combinations significantly increase the net sugar yields compared to the processes applied alone. Using lignin-degrading enzymes also reduces energy consumption, the amount of wastewater, the operational costs of steam explosions, and detoxifies the processed biomass [83,92,94].

However, it should be highlighted that the efficiency of all combined pretreatment methods that involve biological treatment depends strongly on microbial species/strains, culture conditions, biomass type, and the order of pretreatment methods used [83].

Several other combinations of various pretreatments have been studied extensively over the past decade to find the most efficient, economically-viable, and universal solutions, including alkali and metal salt combinations, ultrasound-assisted pretreatment using metal salt with hydrogen peroxide, and a sequential pretreatment comprising of deep eutectic solvents and divalent inorganic salts [92,95,96,97]. However, an ideal method has not been found yet, and further improvement in the pretreatment step is still necessary to overcome the limitations of optimal utilisation of lignocellulosic biomass and make bioethanol production more common and profitable [30,47,56,83,92,98,99,100,101].

### 2.3. Bioethanol Production

After the pretreatment step, bioethanol production from lignocellulosic biomass requires a series of consecutive processes to obtain a final product, including detoxification, hydrolysis, fermentation, distillation, and dehydration [12,47].

#### 2.3.1. Detoxification

Detoxification aims to remove all the toxic compounds from pretreated biomass or hydrolysates, including fermentation inhibitors (such as furan aldehydes, aliphatic acids, and phenolic compounds) that could minimise the enzymes’ efficiency and restrict microbial growth and activity during fermentation. The most common methods to discard inhibitors from biomass and ensure higher bioethanol yield and productivity are, nowadays, various in situ strategies, including membrane extraction, solvent extraction, ion exchange, membrane bioreactors, adsorption, microbial adaptation, using microbial consortium or engineered microorganisms, and several other techniques that are tailored according to pretreatment, hydrolysis, and fermentation methods used in the ethanol production process. Detoxification may be performed separately or integrated into hydrolysis or fermentation [12,102,103].

#### 2.3.2. Hydrolysis

After the pretreatment stage is completed, raw material is subjected to enzymatic hydrolysis. This process is carried out to obtain fermentable sugars, pentoses, and hexoses from polysaccharides present in the pretreated lignocellulosic biomass. Mainly enzymes are employed to catalyse the hydrolysis of cellulose and hemicellulose (xylan), but also acids and alkalis can be used for this purpose (as mentioned in Section 2.2.2) [12,104].

**Figure 6:**
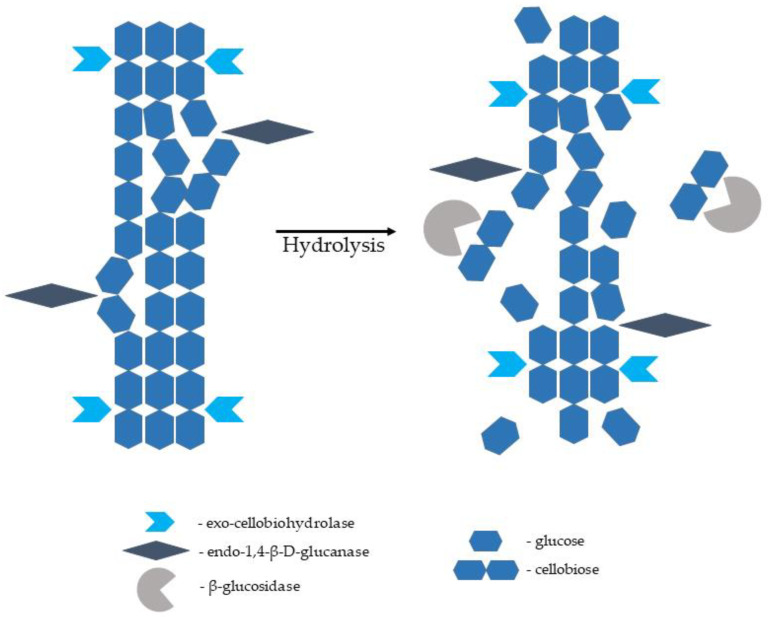
Hydrolysis of native cellulose cellulolytic enzymes (based on [107,110]).

The enzymes capable of hydrolysing cellulose to glucose monomers are known as cellulases. They are multienzyme complexes consisting of mainly three various components, namely endo-1,4-β-D-glucanase (EC 3.2.1.4; breaks intermolecular bonds in cellulose randomly), exo-1,4-β-D-glucanase/exo-cellobiohydrolase (EC 3.2.1.91; removes monomers and dimers from the end of the glucose chain), and β-glucosidase (EC 3.2.1.21; hydrolyses glucose dimers, cellobiose, and other short cellulose oligomers into glucose monomers). Complete hydrolysis of a native cellulose polymer into glucose monomers requires the synergistic action of all three components (Figure 6). Cellulases are sourced from various bacteria and fungi. They are produced by aerobic, anaerobic, mesophilic, and thermophilic microorganisms. Cellulases producing microorganisms include bacterial genera of *Acetovibrio*, *Clostridium*, *Cellulomonas*, *Cellvibrio*, *Bacillus*, *Bacteroides*, *Erwinia*, *Ruminococcus*, *Streptomyces*, and *Actinomycetales* genera of *Microbispora* and *Thermomonospora*. Among fungal species, the most common source of cellulase is *Sclerotium rolfsii* and *Phanerochaete chrysosporium* species, as well as some species belonging to the genera of *Aspergillus*, *Caecomyces*, *Humicola*, *Neocallimastix*, *Oprinomyces*, *Penicillium*, *Schizophyllum*, and *Trichoderma* [105,106,107,108]. Cellulose hydrolysis is difficult because the cellulose microfibrils are stabilised by internal and external hydrogen bonds and surrounded by hemicellulose polysaccharides (mannans and xylans) joined by covalent and hydrogen bonds; hence, the crucial role of the pretreatment stage emerges [104,109].

Since hemicelluloses represent 10–30% of lignocellulosic biomass, their conversion to fermentable sugars is also vital for the high yield of bioethanol. Hemicellulose hydrolysis is easier than cellulose due to its more accessible amorphous structure. On the other hand, its more varied composition and structure, with multiple side chains containing various sugar types, requires a complex set of enzymes. Two groups of enzymes are needed for effective hemicellulose hydrolysis: depolymerising core enzymes that can cleave the backbone and de-branching enzymes (so-called ancillary or auxiliary enzymes) that remove side chains posing steric hindrances to core enzymes, thus increasing the total yield of fermentable sugars obtained from lignocellulosic biomass. The core enzymes include β-1-4-mannosidases (EC 3.2.1.25), endo-1,4-β-mannanases (EC 3.2.1.78), endo-β-1,4-xylanases (EC 3.2.1.8), and xylan 1,4-β-xylosidases (EC 3.2.1.37), while de-branching enzymes are acetylxylan esterase (EC 3.1.1.72), α-L-arabinofuranosidase (EC 3.2.1.55), β-glucuronidase (EC 3.2.1.139), ferulic acid esterase (EC 3.1.1.73), and *p*-coumaric acid esterase (EC 3.1.1-). Similar to cellulases, microorganisms are the source of enzymes for hemicellulose hydrolysis. They include fungi, e.g., *Aspergillus niger*, *Aspergillus awamori*, *Trichoderma reesei*, *Penicillium wortmanii*, *Cochliobacillus carbonum*, *Agaricus bisporus*, and other *Aspergillus*, *Agaricus*, *Trichoderma*, and *Sclerotium* genera, and bacteria, e.g., *Thermotoga maritima*, *Clostridium thermocellum*, *C. cellulovorans*, *Thermobacillus xylanilyticus*, *Paenibacillus polymyxa* cel44C-man26A, *Cellvibrio japonicus*, *Caldibacillus cellulovorans*, *Caldicellulosiruptor Rt*8b, *Caldocellum saccharolyticum*, *Bacillus* spp., and *Streptomyces* spp. The synergistic action of various microbial enzymes ensures high sugar yield from lignocellulosic biomass, thus enhancing bioethanol production [108,111].

The most critical parameters during biomass hydrolysis include solid loading, the concentration of sugars, enzyme loading, the shaking speed, hydrolysis time, the concentration of inhibitors, and the effect of various additives [12,112,113,114].

- Solid loading—High solid loading reduces hydrolysis installation costs and are necessary to obtain syrups with increased sugar concentrations (80–100 g/L), which determines economically viable distillation (i.e., the ethanol concentration in a fermented broth should be above 4% *w*/*w*). It was shown that sugar yield increases with increasing substrate load, but only to some point, after which it decreases. It is mainly because increased cellobiose and glucose concentrations inhibit enzyme activity. Additionally, high solid loading usually translates into a high-viscosity broth, which causes several technical problems due to hampered mixing and impaired mass and heat transfer, affecting the efficiency of enzymes [112,114,115,116,117];
- Enzyme loading—Increased doses of enzymes (or enzyme cocktails) enhance saccharification efficiency providing high glucose yield [12].
- Shaking speed—Optimising shaking/mixing speed is necessary to ensure optimal heat and mass transfer that translates into high glucose yield. Lower speed values result in poor mixing and decreased monosugar yields, while too high of a speed produces shearing forces that may destroy enzymes [117,118,119,120].
- Hydrolysis time—The long time required for complete hydrolysis limits the commercial production of ethanol from lignocellulosic biomass. Therefore, several approaches have attempted to shorten the process by enhancing hydrolysis efficiency, mainly using engineered enzymes/microorganisms or enzyme cocktails and optimising the parameters of the process [121,122].
- Concentration of inhibitors—Inhibitors produced during biomass pretreatment may slow down or even stop enzymatic hydrolysis. Therefore, the detoxification step (see 2.3.1. Detoxification), performed before or during hydrolysis or selecting pretreatment methods producing only a limited amount of inhibitors, is crucial for the process [12,101,102,103,123].
- Effect of various additives—Several different substances were successful as additives in the hydrolysis step to improve glucose yield, including polyethylene glycol (PEG)-based polymers (PEG 600, 4000, 6000), non-ionic surfactants (Tween 80 and Triton X-100), non-catalytic protein (bovine serum albumin (BSA)) or novel chemical surfactants, such as Silwet L-77. Their mode of action is based on blocking the interactions between lignin and enzymes, thus intensifying positive substrate-enzyme interactions and recovering cellulose hydrolysability [124,125,126,127,128,129,130].

Enzymatic saccharification is the most challenging and relatively expensive stage in bioethanol manufacturing from lignocellulosic biomass, with costs estimated at 20–30% of the total production costs. It has also been recognised as a techno-economical bottleneck in the whole process of biomass-to-ethanol bioconversion. Therefore, all crucial steps impacting the yield of fermentable sugars and total bioethanol require careful optimisation while maintaining minimum operational costs to make the production of lignocellulosic ethanol widespread and profitable [1,12,112].

#### 2.3.3. Ethanol Fermentation

**Figure 7:**
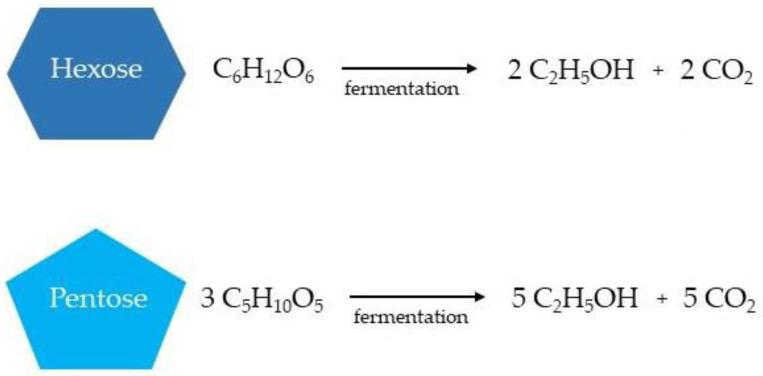
Simplified ethanol production from hexoses and pentoses during the fermentation stage.

In the bioethanol production from lignocellulosic biomass, both hexoses (glucose, fructose, and sucrose) and pentoses are available for ethanol fermentation (xylose, mannose, galactose, and arabinose), resulting in the production of the respective number of ethanol and carbon dioxide molecules (Figure 7) [12,131,132].

For glucose fermentation, industrial strains of *Zymomonas mobilis* and *Saccharomyces cerevisiae* are mainly used, owing to their high ethanol productivity and resistance to high ethanol concentration (up to 120 g/L). However, they are incapable of fermenting pentoses, which limits their use in ethanol production from lignocellulosic raw materials [12,133,134]. Among microorganisms naturally fermenting pentoses are yeasts, such as *Candida shehatae*, *Pachysolen tannophilus*, and *Pichia stipitis* (recently reclassified as *Scheffersomyces stipitis*), and intestinal bacteria; however, the efficiency of the process is minor. Moreover, in the case of pentose-fermenting yeasts, large-scale utilisation is inhibited by their sensitivity to high ethanol concentration (over 40 g/L) and inability to ferment xylose at low pH. In addition, they require microaerophilic conditions and are easily inhibited in the presence of glucose (catabolite repression) and, in a mixed sugar broth, they usually utilise xylose only under glucose-limited conditions [12,135,136,137].

Due to the lack of natural microorganisms for the efficient simultaneous fermentation of pentoses and hexoses, there is a growing interest in using engineering techniques for metabolic processes to construct organisms with the desired characteristics. Metabolic engineering aims to improve microbial activity due to changing enzymatic, transport, and regulatory functions using recombinant DNA technology. It includes analysing metabolic pathways, designing genetic changes, and creating recombinant cells with enhanced desired properties. The modification goal is to obtain a microorganism able to ferment all sugars in the biomass, tolerating stress conditions, showing high resistance to inhibitors, and producing a mixture of synergistic enzymes necessary for the complete hydrolysis of all lignocellulose carbohydrates [46,134,138,139,140]. Among the most frequently modified microorganisms are *Saccharomyces cerevisiae*, *Zymomonas mobilis*, and *Escherichia coli* [46,141,142,143,144], but also other bacterial and fungal species were tested, including *Fusarium oxysporum* [145], *Thermoanaerobacter mathranii* [146], and *Corynebacterium glutamicum* [147]. Designing perfectly engineered microorganisms with the maximum conversion of monomeric sugars and enhanced tolerance to operational conditions will allow for economically feasible industrial production of bioethanol from lignocellulosic biomass [12,46].

Another way to increase the fermentation efficiency is to use immobilised recombinant microbial cells. Immobilisation is placing intact cells on a suitable carrier using entrapment within a porous matrix, adsorption on the solid carrier surface, fixing to the carrier surface by covalent bonding or cross-linking, or encapsulation without altering their preferred catalytic activity. A carrier should be nontoxic, biodegradable, and cost-effective. For yeasts cells, mainly Ca-alginate, carrageenan, cellulose, chitosan, silica-hydrogel, and pre-polymers are used as carriers [12,136,148,149,150,151,152].

The sugar-to-ethanol conversion process can be conducted as a batch, fed-batch, or continuous fermentation, where the fed-batch mode in a stirred tank is the most frequently used in the industry since it provides the optimum conditions required for the microbial strain applied [152,153,154,155].

Industrial biorefineries employ several fermentation technologies to increase ethanol yield and reduce production costs [24,156].

**Figure 8:**
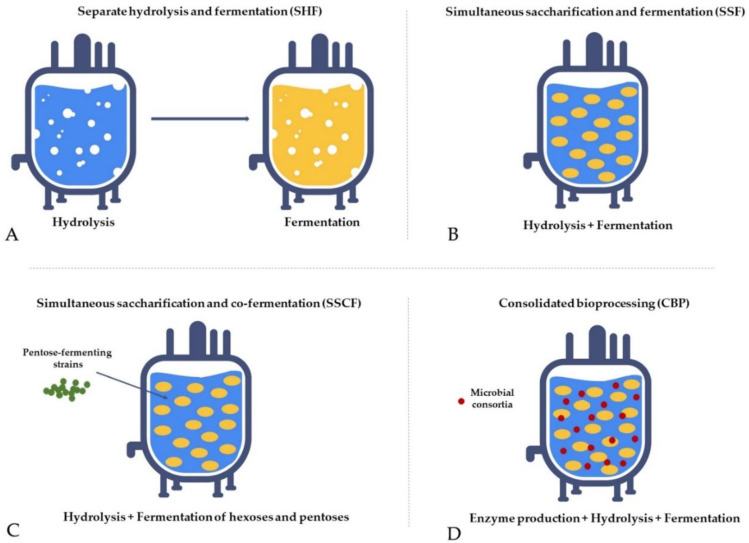
The fermentation strategies used to optimise the process.

- Separate hydrolysis and fermentation (SHF)—Hydrolysis and fermentation processes are conducted independently in different units. Carbohydrates from pretreated biomass are degraded to monosugars in a hydrolysis reactor and subsequently converted to ethanol in a fermentation unit. It is a time-consuming and cost-intensive process due to the long residence time needed for complete hydrolysis, high enzyme loading, and material costs required for two separate units, and its main drawback is end-product inhibition (Figure 8A) [157,158,159,160].
- Simultaneous saccharification and fermentation (SSF)—Hydrolysis and fermentation are carried out in the same unit, which improves hydrolysis rates, yields, and product concentrations compared to SHF due to the continuous removal of the sugars by the yeasts, which reduces the end-product inhibition of the enzyme complex. The main drawback is the difference in optimum temperature between saccharification and fermentation and enzyme inhibition by ethanol, microorganisms, and temperature in the reactor (Figure 8B) [160,161,162].
- Simultaneous saccharification and co-fermentation (SSCF)—Hydrolysis and fermentation are carried out in the same unit with concurrent co-fermentation of pentoses using pentose-fermenting strains, which allows converting both hexoses and pentoses from lignocellulosic biomass, thus increasing ethanol yield. This process is suitable for xylose-rich biomass, such as hardwood and agricultural residues; however, the ethanol yield is lower compared to SSF (Figure 8C) [163,164,165,166].
- Consolidated bioprocessing (CBP)—A single-step process where hydrolysis, fermentation, and enzyme production occur in the same unit. The method employs genetically modified microbes or microbial consortia (e.g., some yeast strains and *Clostridium thermocellum* have already been tested) capable of hydrolysing biomass with enzymes produced on its own and fermenting monosugars to ethanol. The strategy has the potential to revolutionise bioethanol production due to reduced costs for infrastructure and chemicals, making it economically beneficial and environmentally friendly. However, reaching an industrial scale is challenging because of low conversion efficacy, and it still requires further extensive research (Figure 8D) [167,168,169,170].

Effective fermentation of monosugars obtained from lignocellulosic biomass is the next bottleneck in bioethanol production. Several factors might affect its efficiency, including temperature, time, pH, inoculum size, sugar concentration, solid-to-liquid ratio, agitation rate, oxygen content, and rotation speed. Additionally, the operating conditions must be adjusted depending on whether the fermentation is conducted simultaneously or separately with saccharification, which is challenging and requires careful optimisation [12,136,150,171,172].

#### 2.3.4. Distillation and Dehydration

Distillation and dehydration are vital steps for obtaining fuel-grade ethanol from lignocellulosic biomass. Distillation allows for the effective separation of a component substance (such as ethanol) from a miscible liquid mixture (such as fermentation broth) through consecutive selective evaporation and condensation processes based on a difference in their volatilities [173,174,175]. The water content in the post-fermentation mixture is very high, usually exceeding 80% of the dry weight. Therefore, concentrating ethanol up to 96% requires a huge amount of energy, which generates high costs [45]. The first stage of the process is the so-called “drive away the alcohol”. The product (about 37% bioethanol) is then concentrated in a rectification column to a concentration of about 95% and finally dehydrated to a high-quality dry product which holds a minimum of 99.5% ethanol by volume [12,46,176].

Various methods for separating ethanol from a fermentation broth in bioethanol production have been developed, such as adsorption distillation, membrane processes, azeotropic distillation, diffusion distillation, extractive distillation, pervaporation, vacuum distillation, and chemical dehydration, differing in the technique employed, effectiveness and operational costs [17]. Among them, membrane distillation and pervaporation are the most economically viable for bioethanol production.

Membrane distillation is a method that allows for the reduction in the energy expenditure of the process of obtaining ethanol at the stripping stage. During distillation, a membrane separates the fermenting solution from the distillate. Membranes that are used are flat or capillary, porous with gas-filled pores (porosity in the range of 70–85%), hydrophobic (not wetted by liquid), and with high thermal resistance. The process is feasible when there is a pressure difference between molecular components in the gas phase. Different types of membrane distillation have been developed, including contact, air-gap, vacuum, and sweeping gas membrane distillation. The main advantage of using a distillation membrane is the possibility of carrying out the process at a lower temperature. This eliminates the cost of heating the water to the boiling point of ethanol, thus reducing the total costs of bioethanol production. Other advantages of membrane distillation are the possibility of almost 100% retention of non-volatile compounds, lowering the process compared to conventional distillation, obtaining saturated solutions, and implementing durable artificial plastic installations (corrosion-free). Additionally, membrane distillation enables the continuous fermentation process with simultaneous ethanol stripping [17,177,178,179,180].

Pervaporation is another type of membrane process that can be employed for obtaining anhydrous bioethanol on an industrial scale. This process uses the difference in ethanol concentrations on both sides of the asymmetric thick polymer membrane. The separation mechanism is based on the differences in the affinity of ethanol and water to the membrane (dissolving and diffusion capacity) and allows the final ethanol dehydration to be 99.8% [17,181,182,183,184].

## 3. Conclusions

The production of second-generation bioethanol has several benefits in offsetting the general use of fossil fuels by increasing global supplies of liquid transport fuels in response to growing energy demand and improving energy security in regions devoid of fossil resource deposits. Thereby, bioethanol contributes to restricting worldwide dependence on fossil supplies and the petroleum industry, thus helping alleviate the energy crisis. Moreover, the transition from petroleum- to biomass-derived fuels reduces net carbon dioxide emissions per unit of energy produced and used, helping tackle anthropogenic climate change and its consequences for people and the environment.

Lignocellulosic biomass used for bioethanol production seems to be a promising renewable energy source. To avoid conflicts of interest, biorefineries should focus on utilising agro- and industry-waste biomass rather than biomass used for animal feed. In this context, lignocellulosic feedstocks employed as an energy source are particularly beneficial. It is abundant, does not threaten food security, and is inexpensive since it can be derived from native vegetation (e.g., invasive species, forest residues and thinnings, and grass), discarded agricultural residues (corn stove and cereal straw), and even industrial urban waste rich in organic matter.

Unfortunately, the production of cellulosic ethanol is highly challenging due to the complexity and recalcitrance of lignocellulose and the diversity of biomass. It requires several steps to release the energy-carrying carbohydrates from the lignocellulosic complex and convert them into ethanol, starting from biomass pretreatment through hydrolysis and fermentation. These three steps contribute their own unique bottlenecks in the entire production process, seriously affecting the final efficiency of the production process and generating high operating costs. Therefore, intensive research has been conducted to develop new technologies that are efficient, economically viable, and universal for various biomass types, while being environmentally friendly.

Although significant progress has been made in this field in the past decade, including the development of advanced engineered microorganisms or attempts to combine pretreatment, hydrolysis, and fermentation, or part of them into a single, more efficient step, there are still several gaps between novel findings and practical applications. Some of the most crucial challenges include the following:

- the selection of a suitable pretreatment strategy that is cost-effective and does not impede the overall efficiency of enzymatic saccharification,
- the improvement of the anaerobic digestibility of biomass,
- limiting carbohydrate degradation and the generation of inhibitors during pretreatment to prevent conversion yield loss,
- downsizing the consumption of toxic chemicals, as well as energy and water,
- the improvement and application of novel biocatalysts that can enhance the efficiency of the saccharification process,
- increasing the efficiency of individual enzymes by designing enzymes with enhanced specific activity, thermal stability, and reduced end-product inhibition, and
- reducing the overall footprint of the process.

Detailed knowledge about the structure and composition of different biomass types is required, as well as the effects of individual pretreatment techniques on various biomass materials at the macro and molecular scales. Additionally, a thorough study of the interactions between biomass, microorganisms, products, and by-products generated during hydrolysis and fermentation at the molecular scale is necessary to establish optimal conditions for those processes. The existing knowledge is broad, but even more comprehensive interdisciplinary research is still needed to bring bioethanol production into a profitable and pervasive light for commercial use. However, it should also be remembered that transitioning from a laboratory to a commercial scale is extremely difficult and requires additional pilot-scale studies with optimisation and high financial expenditure.

As for now, it seems that just using lignocellulosic biomass as a sustainable feedstock for bioethanol production does not guarantee a successful transition from petroleum-based to renewable biomass-derived energy. It seems that the strategy to utilise all components of the lignocellulosic complex by employing cost-competitive manufacturing processes designed with green chemistry is more likely to succeed. The future of this energy sector will be integrated biorefineries that produce both energy and value-added components for the chemical industry based on green chemistry principles with respect to the environment. This is achievable through enhancing the efficiency of all used materials and energy, reducing waste production and toxicity, and reusing resources and by-products. Integrated biorefineries are gaining interest worldwide as they support the circular bioeconomy concept. However, greener processes and technologies are required, such as employing water-based reactions and environmentally friendly oxidants instead of materials and chemicals with high environmental burdens, or those using alternative energy-saving pretreatment methods, such as ultrasound or microwaves, which require time, effort, and financial investment.

Since the 1970s, tremendous progress has been made to alleviate the use of fossil fuels. With the persistent passion of researchers worldwide, there is great optimism for the future of bioethanol from lignocellulosics. This review is meant to not only educate on bioethanol processes and their challenges, but also to illuminate novel and debatable research ideas that will tackle these challenges and build sustainable partnerships in an interdisciplinary fashion to combat the global energy crisis at hand.

## Figures and Tables

**Table 1:** The main fuel ethanol producers in the European Union (in millions of litres per year) [23].

Country/Calendar Year	2014 ^r^	2015 ^r^	2016 ^r^	2017 ^r^	2018 ^r^	2019 ^e^	2020 ^e^	2021 ^f^
France	1018	1039	987	1000	1138	1299	1049	1095
Germany	920	870	882	810	799	676	875	950
Hungary	456	591	633	633	645	689	639	640
Netherlands	519	563	443	532	563	570	538	570
Spain	454	494	328	377	522	547	487	480
Belgium	557	557	570	620	646	620	380	380
Poland	181	214	241	258	259	286	277	285
Austria	230	223	224	235	251	254	241	255
United Kingdom	329	538	658	684	443	190	127	190
**Total**	**5190**	**5165**	**5159**	**5373**	**5497**	**5281**	**4747**	**5000**

r = revised/e = estimated/f = forecasted EU FAS Posts. Source: EU FAS Posts (based on [23]).

**Table 2:** Second-generation or mixed biorefineries in the US (state as of 2022) [27]; MGY–million gallons per year; biorefineries under construction are in italics.

Company	City	State	Feedstock	Production Capacity (MGY)	Under Construction (MGY)
*NewEnergyBlue LLC*	Mason City	IA	Cellulosic Biomass	-	20
Project LIBERTY	Emmetsburg	IA	Cellulosic Biomass	25	-
*VERBIO North America Corp.*	Nevada	IA	Corn/Cellulosic Biomass	-	60
Quad County Corn Processors	Galva	IA	Corn/Cellulosic Biomass	38	-
Ace Ethanol LLC	Stanley	WI	Corn/Cellulosic Biomass	54	-
POET Biorefining-Iowa Falls LLC	Iowa Falls	IA	Corn/Cellulosic Biomass	115	-
Louis Dreyfus Grand Junction LLC	Grand Junction	IA	Corn/Cellulosic Biomass	125	-
POET Biorefining-Shell Rock LLC	Shell Rock	IA	Corn/Cellulosic Biomass	140	-
PureField Ingredients LLC	Russell	KS	Corn/Sorghum/Cellul. Biomass	55	-
Pelican Acquisition LLC	Stockton	CA	Corn/Sorghum/Cellul. Biomass	60	-
ELEMENT LLC	Colwich	KS	Corn/Sorghum/Cellul. Biomass	70	-
*LanzaTech Freedom Pines Fuels LLC*	Soperton	GA	Industrial Off-Gases/Biomass/Biogas	-	10
Total	-	-	-	682	90

**Table 3:** The operational or close to operational advanced biofuel plants in the EU that produce second-generation bioethanol commercially (state as of 2022, based on [23]).

Country	Feedstock	Capacity (Million Litres Per Year)	Year of Opening
Finland	Sawdust	10	2018
Italy	Biomass	28	2020
Austria	Wood sugar	30	2020
Romania	Wheat straw	65	2021
Bulgaria	Corn stover	50	2021
Total	-	183	-

Based on: EU FAS Posts [23].

## Data Availability

Not applicable.
